# Clinical benefit of faecal microbiota transplantation administered *via* a single retention enema as an adjunctive treatment in dogs with chronic enteropathy: a randomised controlled trial

**DOI:** 10.1111/jsap.70137

**Published:** 2026-04-17

**Authors:** F. Allerton, M. J. Whittle, L. Durkin, C. Prior, M. Trehy, H. Swales, F. Duplan, S. Borgonovi, G. Pinchbeck, I. Gajanayake, M. Dunning, T. Sparks, P. Watson, G. C. A. Amos, K. McCallum, J. Bazelle, A. Kent

**Affiliations:** ^1^ Willows Veterinary Centre & Referral Service Solihull UK; ^2^ Waltham Petcare Science Institute Melton Mowbray UK; ^3^ London Vet Specialists London UK; ^4^ Bridge Referrals Boldon Colliery UK; ^5^ Langford Vets Langford UK; ^6^ DWR Veterinary Specialists Six Mile Bottom UK; ^7^ Institute of Infection, Veterinary & Ecological Sciences University of Liverpool Leahurst UK; ^8^ School of Veterinary Medicine and Science University of Nottingham Sutton Bonington UK; ^9^ Department of Veterinary Medicine University of Cambridge Cambridge UK; ^10^ Davies Veterinary Specialists Higham Gobion UK; ^11^ Blaise Veterinary Referral Hospital Birmingham UK

## Abstract

**Objectives:**

To evaluate the clinical benefit of faecal microbiota transplantation administered *via* a single retention enema, as an adjunctive treatment in the management of dogs with chronic enteropathy.

**Materials and Methods:**

Blinded, randomised controlled trial. Dogs with chronic enteropathy (>3 weeks of small or mixed intestinal diarrhoea) were randomly allocated to either the faecal microbiota transplantation or standard treatment group (ratio 1:1) *via* blinded selection. Dogs in the standard treatment group had a diet change only, while dogs in the faecal microbiota transplantation group had a diet change and faecal microbiota transplantation. faecal microbiota transplantation was performed using fresh faecal material from donor dogs, screened for selected enteropathogens and administered *via* retention rectal enema. Outcomes measured included the Canine Inflammatory Bowel Disease Activity Index, faecal score and the owner's reported improvement. Group comparisons were made using Fisher's exact tests (owner‐reported outcomes) and Kruskal–Wallis tests adjusted for ties (Canine Inflammatory Bowel Disease Activity Index and faecal score).

**Results:**

Forty‐two dogs with chronic enteropathy (median Canine Inflammatory Bowel Disease Activity Index score 6 [range 4 to 11]) were included in the study. Twenty‐five dogs were randomly assigned to receive faecal microbiota transplantation, while 17 dogs were allocated to standard treatment. A progressive improvement in stool consistency (reduced faecal score) was recorded over time for most dogs in both groups. By Day 90, the rates of owner‐defined clinical improvement were 76% (CI 54% to 90%) in the faecal microbiota transplantation group and 73% (CI 40% to 92%) in the standard treatment group. No significant differences were evident between the two groups based on the proportion of owners that reported clinical improvement, Canine Inflammatory Bowel Disease Activity Index score or faecal score.

**Clinical Significance:**

This study did not demonstrate a clear clinical benefit for adjunctive faecal microbiota transplantation *via* single retention enema in dogs with chronic enteropathy compared to diet change alone, although the small sample size means that a type II error cannot be excluded. The similar outcome for both groups supports high rates of food responsiveness among this cohort of chronic enteropathy dogs.

## INTRODUCTION

Canine chronic enteropathies have been described as food‐responsive (FRE), antibiotic‐responsive (ARE), immunosuppressant‐responsive (IRE) or non‐responsive (NRE) (Dandrieux, 2016), with diet, antibiotic or immunosuppressant therapeutic trials used to facilitate differentiation between categories (Allenspach et al., 2016). However, this clinical classification relies on causal inference, introducing a substantial subjective judgement, which may lead to incorrect attribution of treatment effect. Clinical improvement has been reported more commonly for FRE compared to ARE or IRE, reflecting differences in case severity (Allenspach et al., 2016; Dandrieux & Mansfield, 2019; Makielski et al., 2019) and, due to overlapping clinical features, possible misalignment of therapy with the underlying pathology.

A defined mechanism for antibiotic responsiveness in chronic enteropathy has not been established. Putative enteropathogens were not identified in 14 tylosin‐responsive dogs, challenging a direct antibacterial role (Westermarck et al., 2005). Furthermore, both tylosin and metronidazole have been shown to provoke dysbiosis when administered to healthy dogs, refuting a beneficial influence on the microbiome (Igarashi et al., 2014; Manchester et al., 2019; Suchodolski et al., 2009). Proposed anti‐inflammatory or immunomodulatory actions of metronidazole (Shakir et al., 2011) and tylosin (Cao et al., 2006) have been cited by veterinarians as justification for their use (Makielski et al., 2019; Ng et al., 2025). However, no additional benefit was derived when metronidazole was used in combination with prednisolone compared to prednisolone alone (Jergens et al., 2010). The use of antibiotics for non‐antimicrobial effects is discouraged (Weese et al., 2015).

The growing risk to human and animal health from multi‐drug resistant bacteria (Antimicrobial Resistance Collaborators, 2022; O'Neill, 2016; Weese et al., 2015) is a stark reminder of the need to ensure a rational approach to antimicrobial use. Amoxicillin administration to healthy dogs led to a transiently increased expression of resistance in faecal *Escherichia coli* (Grønvold et al., 2010). Consideration of the relative benefits and harms from antibiotic therapy has prompted a re‐evaluation of antibiotic trials within the chronic enteropathy treatment algorithm (Cerquetella et al., 2020), leading to the pursuit of alternative approaches that seek to support, rather than disrupt, the microbiome. Indeed, a recent review has proposed a revised name, microbiota‐related modulation responsive enteropathy (MrMRE) to replace the ARE category advocating the use of prebiotics, probiotics, postbiotics, synbiotics or faecal microbiota transplantation (FMT) to positively manipulate the microbiome (Dupouy‐Manescau et al., 2024).

In human medicine, FMT is recommended for the treatment of recurrent *Clostridioides difficile* infection (Mullish et al., 2024). It may also help manage some chronic gastrointestinal disorders, including ulcerative colitis (Haifer et al., 2022; Kedia et al., 2022). A Cochrane metanalysis could not determine a benefit of FMT for inducing or maintaining remission in people with Crohn's disease (Imdad et al., 2023). In dogs with chronic enteropathy at varied stages of disease management, a randomised clinical trial (Collier et al., 2022), a longitudinal prospective study (Vecchiato et al., 2025), two case reports (Niina et al., 2019; Sugita et al., 2021) and several preliminary studies (Cantas et al., 2025; Niina et al., 2021; Pérez‐Accino et al., 2025; Toresson et al., 2023) have documented the response to FMT administered *via* rectal enema with generally favourable results (variable outcome measures). However, other treatments were often concurrently administered and only 1 study included a control group (Collier et al., 2022), so the real clinical benefit of FMT remains uncertain. Mild adverse effects attributable to FMT including vomiting and inappetence, were reported following administration to healthy dogs (Lee et al., 2024).

The primary objectives of this multi‐centre, blinded, randomised controlled study were to compare outcomes after 7 and 28 days of either standard treatment (*i.e*. a diet trial with either a hydrolysed or novel‐protein source diet) or standard treatment and adjunctive FMT administered by single retention enema, in dogs with chronic enteropathy. The authors hypothesised that a greater proportion of the FMT group would demonstrate clinical improvement at each timepoint.

## MATERIALS AND METHODS

This trial was a blinded, randomised control trial based in six referral veterinary clinics across the UK designed in accordance with the CONSORT guidelines. Ethical approval was obtained from the University of Nottingham (ref. 3025 191111) and the Waltham Petcare Science Institute Animal Welfare and Ethics Review Board. Further, an Animal Test certificate was obtained from the Veterinary Medicines Directorate (ATC‐S‐133), renewed after 2 years.

### Case enrolment

Privately owned dogs, between 6 months and 10 years of age, with a 3‐week or longer history of small intestinal or mixed (small and large intestinal) diarrhoea were recruited to the study. Dogs were excluded if they had exclusively large intestinal diarrhoea, were significantly hypoalbuminaemic (serum albumin <20 g/L), had any extra‐intestinal cause of diarrhoea including hypoadrenocorticism, as evidenced through blood tests or diagnostic imaging, or if they had recently (previous month) been treated with a probiotic or any immunosuppressant medication. The original protocol (exclusion of animals that had received an antibiotic treatment within the previous month) was altered after 1 month to allow inclusion of dogs that had been recently treated with antibiotics. Inclusion and exclusion criteria are summarised in Tables S1 and S2.

The trial was outlined to pet owners at the initial consultation. Informed consent was acquired for any potential participant. The owner also had to accept to be blinded as to whether their dog received FMT. All dogs underwent a thorough physical examination, collection of a blood sample for biochemistry and haematology profiles, measurement of cobalamin and basal cortisol concentrations (unless such samples had been obtained in the 2 weeks prior to presentation) and abdominal imaging *via* either ultrasonography or computed tomography (CT) performed under sedation (protocol to be determined by the case clinician). All diagnostic imaging was reported by a European specialist in diagnostic imaging or was overseen by a European specialist in internal medicine. Additionally, an adrenocorticotropic hormone (ACTH) stimulation test was performed in any dog with a basal cortisol below the threshold to exclude hypoadrenocorticism (≤55 nmol/L). Dogs were fasted for at least 12 hours prior to sedation.

Immediately following the completion of abdominal imaging, eligible dogs were randomly assigned to either the FMT treatment or standard treatment group (ratio 1:1) *via* blinded selection of a numbered card from an envelope (even numbers were assigned to the FMT treatment group). A change of diet was recommended for all dogs in the study. The diet selected depended on the individual diet history of each dog but was either an alternative hydrolysed protein diet or novel‐protein diet and in all cases represented a diet change. Cobalamin supplementation was recommended for all dogs with serum cobalamin <400 ng/L, administered parenterally or orally according to the preferences of the case clinician and owner. Additional treatments were administered at the discretion of the case clinician.

### Faecal donor eligibility criteria

Donor dogs (*n* = 9) were aged 7 years or less and owned by employees at participating veterinary centres, who provided informed consent for the use of their dog's faeces and data. Donors were required to have up‐to‐date vaccinations, receive regular prophylactic endoparasiticide treatment and be fed a nutritionally complete balanced commercial diet with no raw food component. Donor dogs were required to have had no history of gastrointestinal clinical signs (vomiting or diarrhoea) nor to have received any antibiotic treatment in the previous 12 months. All donor dogs were in apparent good health upon physical examination (BCS 4 or 5/9) and were screened for *Salmonella* spp., *Coccidia* spp. by faecal culture and for *Giardia* spp. *via* faecal antigen assay^1^ at a single veterinary diagnostic laboratory prior to inclusion.

### FMT procedure

Fresh faecal material was collected on the day of administration of FMT. Faecal material was made available for all eligible dogs but only administered to those assigned to the treatment group. Case clinicians were not blinded to treatment group since the veterinarian performed the FMT procedure. Study participants received FMT under the sedation used for diagnostic abdominal imaging. The fresh faecal material was weighed, diluted in a ratio of 1:4 with sterile saline (0.9%) and then blended with an electric blender. The suspension was filtered through a sieve with the filtered material aspirated into 50 mL sterile syringe(s). A volume of 10 mL/kg of transplant material (equivalent to 2 g of faeces/kg bodyweight of recipient) was administered rectally *via* a 16 French Foley catheter inserted approximately 20 to 25 cm into the rectum and bulb inflated. The material was kept in situ for 20 minutes (retention enema) before the dog was recovered routinely from sedation. Each veterinarian who administered FMT treatment was responsible for ensuring that the dog's owners remained blinded to treatment group during the outpatient debrief and participant collection from the clinic. Participants were removed from the trial if the owner became aware of the treatment group or if the owner requested to be withdrawn at any point. All owners were told to treat their pet as if they had received FMT treatment (instructed to minimise physical exercise and withhold food for a further 4 to 6 hours).

### Baseline assessment

Baseline assessments were conducted at the initial consultation (Day 0) and recorded into the electronic data capture (EDC) platform (Castor^®^) by the veterinarian. Recorded details included signalment information (age, sex, neuter status and breed), bodyweight, body condition score (9 point scale),^2^ recent antibiotic history, Canine Inflammatory Bowel Disease Activity Index (CIBDAI) (Jergens et al., 2003), diet history, results of blood testing (serum albumin; total protein; cholesterol; creatinine; total bilirubin; alanine transferase activity; haematocrit; total leukocyte (WBC), neutrophil, lymphocyte and eosinophil counts; cobalamin; folate (if available); trypsin like immunoreactivity (if available); and canine pancreatic lipase immunoreactivity or DGGR lipase activity (if available); basal cortisol) and imaging findings.

### Subsequent visits

Direct follow‐up by the case veterinarian was collected on Days 7, 28 and 90, in person or by telephone. Eligibility to remain on the trial was reassessed by the veterinarian with any participants that deviated from the pre‐defined treatment protocol withdrawn from the trial. Information collected included owner‐reported clinical signs (components of the CIBDAI), whether any additional treatment had been administered and whether the diet had been altered since previous contact. Dog owners were also tasked to record CIBDAI scores and faecal consistency according to the WALTHAM^™^ Faeces Scoring System^3^ on designated days within 90 days following enrolment. This information was periodically collected from pet owners *via* an automated survey emailed to the owners from the EDC platform on Days 2, 7, 28, 60 and 90.

Participants that presented persistent clinical signs of chronic enteropathy at Day 28 were offered unblinded FMT as part of the study regardless of their study group, at the discretion of the recruiting veterinarian. However, no further clinical data from these dogs were included in study data analysis. Any adverse events observed during the study were recorded in the EDC.

### Statistical analysis

#### Sample size calculation

The primary outcome measures, to assess the clinical efficacy of FMT treatment were defined, a priori, as a 2‐point change from baseline in CIBDAI score at Days 7 and 28. Faecal scores were assessed as secondary outcome measures from Days 1 to 28, 53 to 60 and 83 to 90. Mean faecal scores and number of stools were calculated for each dog on each assessment day. A subjective evaluation of improvement by the dog owner (yes or no) was included as an additional outcome.

A binary outcome superiority trial power calculation was conducted to determine the sample size for the study. A total sample size of 118 dogs (59 per group) was targeted to provide an 80% chance of detecting, at a 5% significance level, an improvement in the primary outcome measure for 70% of the dogs in the standard treatment group and 90% in the FMT treatment group. New case recruitment was closed after 4 years.

Continuous variables are summarised by median and range. Signalment variables and CIBDAI on Day 0 of the two groups were compared using Mann–Whitney tests adjusted for ties. The proportion of dogs reported by owners as having improved, and the proportion of dogs with a reduction of CIBDAI from baseline of 2 or more at Days 7 and 28, were compared between the two groups using Fisher's exact tests. These two variables were analysed as linear mixed models with dog as a random effect and day (as a continuous variable), group and day × group interaction as fixed effects. Changes in CIBDAI from baseline were compared between 10 categories (nine donor dogs and the standard treatment) using Kruskal–Wallis tests adjusted for ties. Analysis was undertaken in Minitab21. Significance was considered as *P* < .05.

## RESULTS

### Animals

Owners of dogs with compatible clinical signs, indicating an interest in participating in the study at initial consultation, were eligible for inclusion. Dogs were excluded if they had a serum albumin <20 g/L (*n* = 2) or if arrangements could not be made to have faecal material available at the time of group assignment (*n* = 1). Forty‐two dogs, aged 8 to 115 months (median 30 months) matched the inclusion criteria and were enrolled from 6 referral centres (reporting 24, 12, 3 and 1 case (three centres) each). There were 13 cross‐breed dogs, four German Shepherds, two each of boxer, cocker spaniel, golden retriever, Hungarian Vizsla, Labrador retriever, miniature schnauzer and springer spaniel and 11 other breeds with one representative (Table S3). Twenty‐seven dogs (64%) were male, of which 17 were intact and 10 castrated, and 15 dogs (36%) were female, of which seven were entire female dogs and eight were spayed. The median weight of dogs was 16.3 kg (range 4.4 to 42.4 kg) and the median body condition score was 4 (range 2 to 7).

At presentation, changes in activity levels were reported in 19 dogs (7 moderate and 12 mild changes); in appetite in 22 dogs (1 severe, 8 moderate and 13 mild changes); vomiting was reported in 23 dogs (1 moderate and 21 mild changes); altered stool consistency in 42 dogs (13 severe, 23 moderate and 6 mild changes); increased frequency of defecation in 37 dogs (1 severe, 14 moderate and 22 mild changes); and weight loss in 28 dogs (1 severe, 11 moderate and 16 mild changes).

The median serum albumin value was 30 g/L (range 20 to 39 g/L). Twenty‐seven of 42 dogs (64%) had a basal cortisol ≥55 nmol/L. The remaining 15 dogs had a basal cortisol <55 nmol/L and so had an ACTH stimulation test performed that excluded hypoadrenocorticism. Other haematological and biochemical values are summarised in Table S4.

Abdominal imaging was performed by ultrasound in 40 dogs (95%) and by CT (with gastrointestinal‐focused ultrasound) in two dogs. No abnormalities were detected in 23/42 imaging studies (55%). Of the 19 dogs with ultrasound changes, 12 dogs (29%) had changes related to the GI tract due to GI wall thickening (*n* = 6), GI wall thickening and mucosal speckles (*n* = 4) or mucosal speckles only (*n* = 2). Nine dogs with GI wall thickening had thickening of the muscularis layer (*n* = 6), submucosa (*n* = 1) or mucosa (*n* = 2). Nine dogs (21%) had jejunal lymph node enlargement, two dogs had hepatic, one dog medial iliac and one dog colic lymph node enlargement. Lymph node appearance was described as hyperechoic (*n* = 4), hypoechoic (*n* = 2) or normal (*n* = 4). One dog had an enlarged and hyperechoic liver, another had a hyperechoic liver and a third had a hypoechoic liver; these changes were not associated with biochemical evidence of hepatic disease. No dogs had any changes seen relating to the pancreas. One dog had a missing left kidney and caudal vena cava duplication. Two dogs had small splenic nodules, and one dog had a bladder urolith.

### Group assignment

Twenty‐five dogs (60%) were randomly assigned to receive FMT, while 17 dogs (40%) were allocated to standard treatment only. There were no significant differences between groups in terms of age (*P* = .324), weight (*P* = .798) or body condition score (*P* = .640) at Day 0 (Table 1). The FMT group consisted of 17 male dogs (nine entire, eight neutered) and eight female dogs (four entire, four neutered). The standard treatment group consisted of ten male dogs (eight entire, two neutered) and seven female dogs (three entire, four neutered). Median CIBDAI score at presentation was 6 (range 4 to 11) in the FMT group and 6 (range 4 to 9) in the standard treatment group, which was not significantly different (*P* = .694).

**Table 1:** Comparison of signalment at Day 0 in dogs receiving faecal microbiota transplantation (FMT, *n* = 25) with those on standard treatment (*n* = 17)

Variable	Group	*N*	Median	Minimum	Maximum
Age (years)	FMT	25	2.7	0.7	9.6
	Standard	17	2.5	0.7	9.0
Weight (kg)	FMT	25	16.7	4.4	42.4
	Standard	17	13.5	5.3	43.0
Body condition score (9‐point scale)	FMT	25	4.0	2.0	7.0
	Standard	17	4.0	3.0	6.0

Two dogs (5%) were withdrawn from the study before Day 28. One dog (standard treatment) was diagnosed with exocrine pancreatic insufficiency and started on pancreatic enzyme supplementation, and another dog (FMT) was withdrawn after the owner requested to be unblinded. A further 22 additional treatments were administered to 16 other dogs during the 90 days of the trial (Table 2). Outcome analysis was performed for the 40 eligible dogs and for censored data groups (with dogs excluded from the time of administration of any of the following medications: probiotics, antibiotics, proton pump inhibitors or systemic corticosteroids).

**Table 2:** Adjunctive medications prescribed during the 90‐day trial period in dogs receiving faecal microbiota transplantation (FMT) or on standard treatment

Medication	FMT				Standard treatment				Total
	D0	D7	D28	D90	D0	D7	D28	D90	
Amoxicillin‐clavulanate (for urinary tract infection)			1						1
FMT			1				1		2
Fusidic acid (topical)			1						1
Maropitant	2				1				3
Metoclopramide								1	1
Omeprazole					2				2
Levetiracetam			1						2
Paracetamol		1							2
Phenylpropanolamine							1		2
Prednisolone				1		1*	2		4
Probiotics	1	3		1	2				7
Total	3	4	3	2	5	1	3	1	24

* No further data was collected for this dog after D7

Repeat (or first) FMT was offered to 12 dogs that were reported to have not improved (CIBDAI unchanged or increased) at the Day 28 check‐up. Ten owners declined FMT at that stage. FMT was performed in one dog originally on standard treatment at Day 40 due to persistent clinical signs. No response was observed in this dog and clinical signs were still apparent after 90 days. FMT was performed at Day 30 in one dog originally in the FMT group as the initial clinical improvement had deteriorated after 2 weeks with a recurrence of diarrhoea. A transient improvement in stool consistency was seen, again followed by recurrence of diarrhoea; clinical signs resolved later following a further diet change. No adverse effects attributable to FMT were reported in any of the dogs administered FMT at any stage of the study besides a failure to improve.

### Clinical response (owner‐reported improvement)

No significant differences (*P* > .05) between the two groups were evident based on the proportion of owners that reported clinical improvement on Days 7, 28 and 90 (Table 3). This applied both to analysis of all dogs and the groups censored following the administration of other medications.

**Table 3:** Clinical response (% owner‐reported improvement) on Days 7, 28 and 90 in dogs receiving faecal microbiota transplantation (FMT) or on standard treatment

	FMT		Standard		*P* value	
	All dogs	Censored data	All dogs	Censored data	All dogs	Censored data
Day 7	14/23 (61%)	14/22 (64%)	11/16 (69%)	8/12 (67%)	.740	1.000
Day 28	17/22 (77%)	15/18 (83%)	10/14 (71%)	8/11 (73%)	.712	.646
Day 90	16/21 (77%)	12/15 (80%)	8/11 (73%)	6/7 (86%)	1.000	1.000

Analyses are performed for all study‐eligible dogs and for censored data (where dogs administered corticosteroids, proton pump inhibitors, antibiotics or probiotics have been excluded)

### Clinical response (CIBDAI)

**FIG 1:**
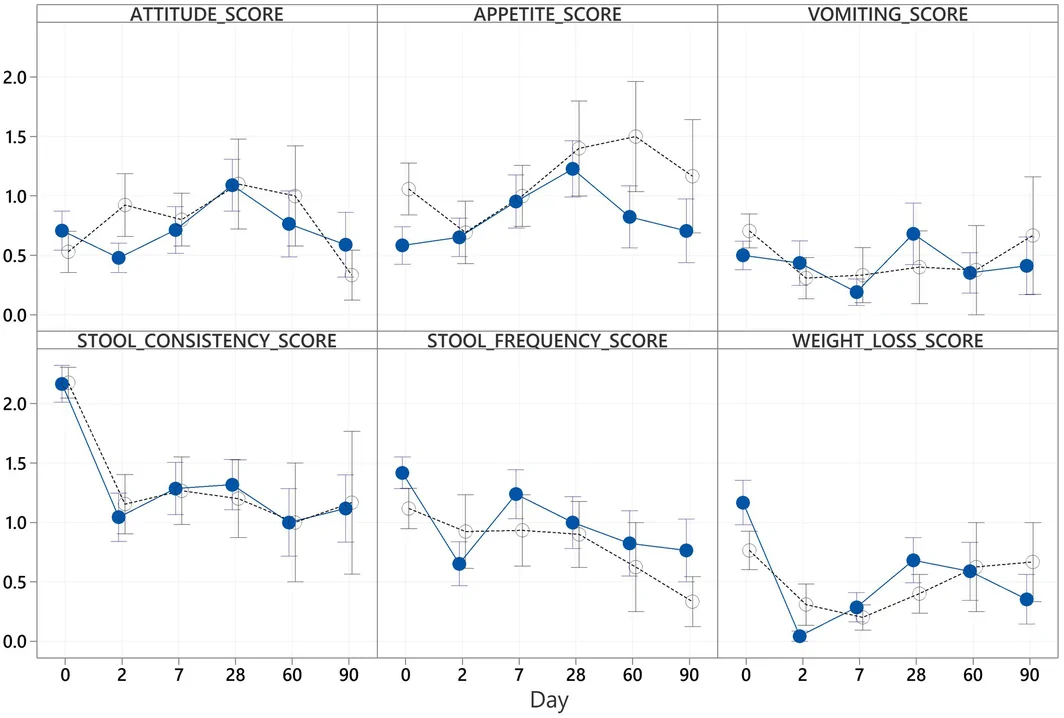
The individual component scores that constitute the Canine Inflammatory Bowel Disease Activity Index (CIBDAI) for dogs receiving faecal microbiota transplantation (FMT, solid symbols) with those on standard treatment (open symbols). Means and one standard error bars are shown. Please note the non‐linearity in the horizontal axis.

**FIG 2:**
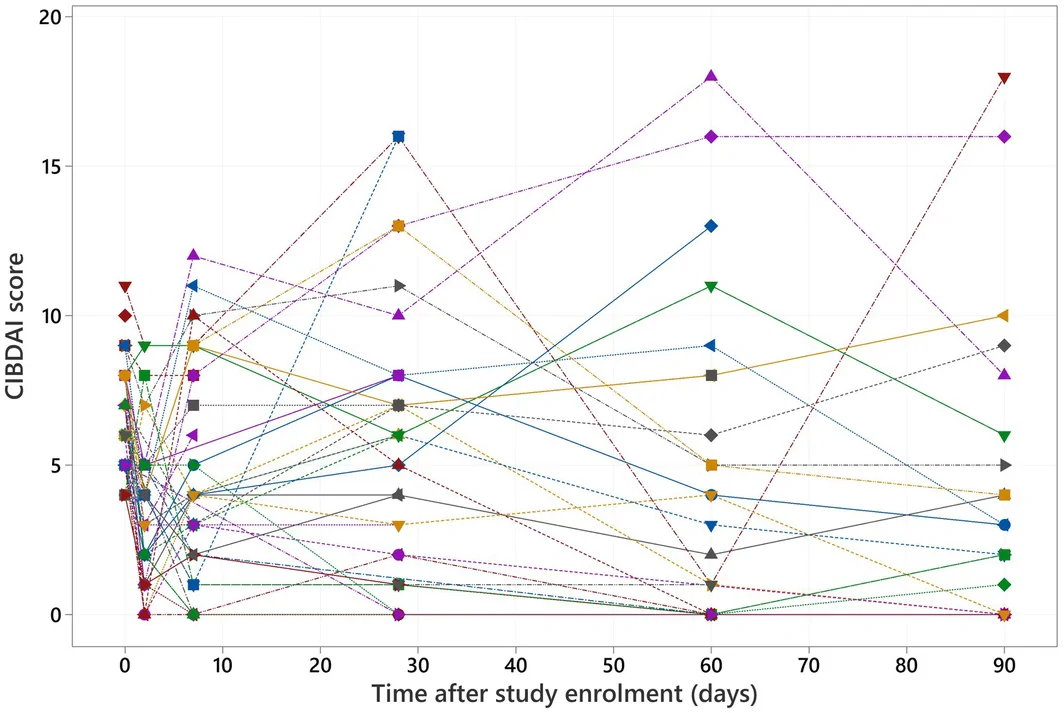
Scatterplot tracking Canine Inflammatory Bowel Disease Activity Index (CIBDAI) for all dogs recruited into the study with measures recorded at Days 0, 2, 7, 28, 60 and 90 after enrolment. Symbols represent different dogs but are often merged when they overlap.

A clinical response, categorised as a reduction in the CIBDAI of 2 or more compared to Day 0, was reported in 18/34 (53%) dogs at Day 7 and 14/30 (47%) dogs at Day 28 regardless of treatment group. There was no significant difference in response rate between groups (with or without censored data) at these or other time points; however, the largest difference was observed on Day 2 when comparing all dogs and at Days 2 and 90 when considering only the censored data. Response rates for both groups at all time points are shown in Table 4. The difference in individual component scores that constitute the CIBDAI is shown for the two groups (all dogs) at each time point in Fig 1 and tracked for individual dogs across the 90‐day surveillance period (Fig 2).

**Table 4:** Responses (reductions in CIBDAI from baseline of 2 or more) at measured time points in dogs receiving faecal microbiota transplantation (FMT) or on standard treatment

	FMT		Standard treatment		*P* value	
	All dogs	Censored data	All dogs	Censored data	All dogs	Censored data
Day 2	18/22 (82%)	18/21 (86%)	7/12 (58%)	6/10 (60%)	.224	.172
Day 7	11/20 (55%)	10/19 (53%)	7/14 (50%)	6/11 (55%)	1.000	1.000
Day 28	10/21 (48%)	8/17 (47%)	4/9 (44%)	3/8 (37%)	1.000	1.000
Day 60	11/17 (65%)	9/12 (75%)	5/8 (62%)	4/7 (57%)	1.000	.617
Day 90	14/17 (82%)	11/13 (85%)	4/6 (67%)	3/5 (60%)	.576	.533

Analyses are performed for all study‐eligible dogs and for censored data (where dogs administered corticosteroids, proton pump inhibitors, antibiotics or probiotics have been excluded). *P* values determined by comparison of groups using Fisher's exact tests

### Clinical response (faecal score)

**FIG 3:**
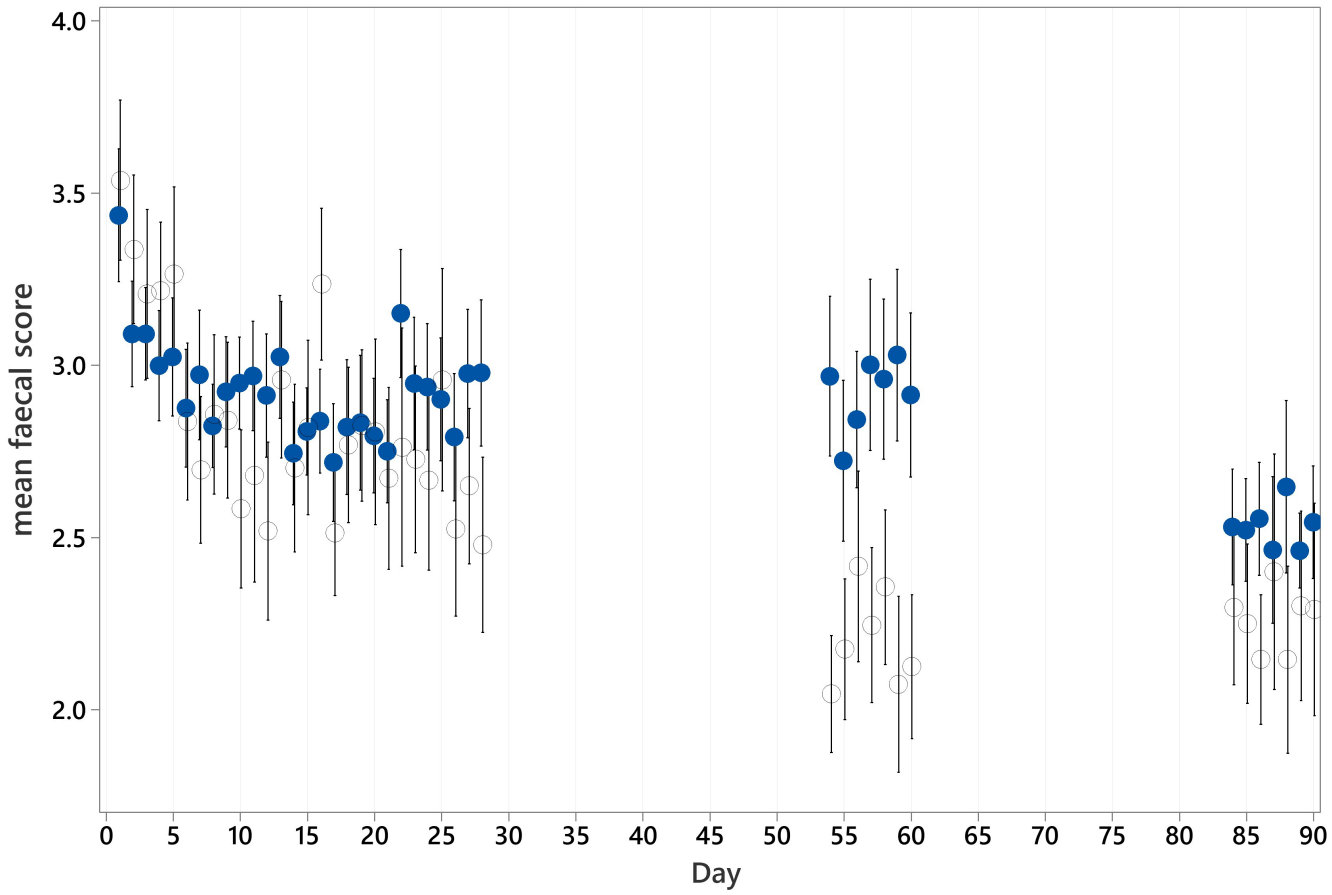
Mean faecal score ± 1 standard error for dogs receiving faecal microbiota transplantation (FMT, solid symbols) and those on standard treatment (open symbols).

A progressive improvement in stool consistency (reduced faecal score) was recorded over time (significant day effect (*P* < .001)) as shown in Fig 3. Considering all collected data the group effect was not significant (*P* = .925), but a significant day × group interaction was found (*P* < .001) with the standard treatment group having a significantly more negative trend, that is towards improved consistency.

**FIG 4:**
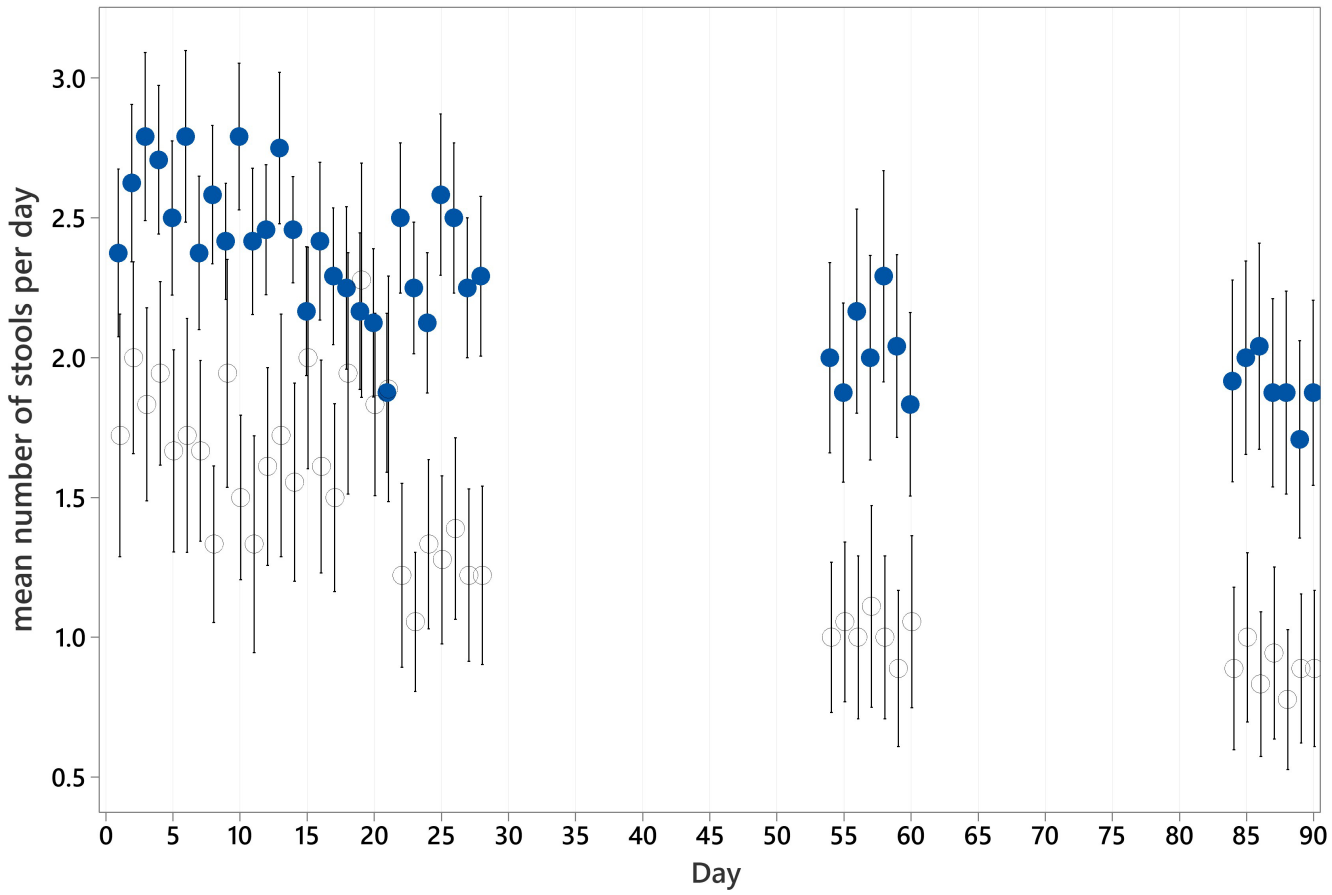
Mean number of stools per day ±1 standard error for dogs receiving faecal microbiota transplantation (FMT, solid symbols) and those on standard treatment (open symbols).

FMT‐treated dogs produced significantly more stools per day (*P* = .019) compared to dogs receiving standard treatment (Fig 4), which was apparent even at Day 0. Both groups had a significant reduction with time (*P* < .001), but the day × group interaction was not significant (*P* = .066).

### Donor effect

Nine different donor dogs were used (from 1 to 8 times) to provide the material for the 25 initial FMT procedures. There was no significant difference in responses to any specific donor on any of the five outcome days (all *P* > .201).

The dataset was considered too small to evaluate the potential influence of other factors (*e.g*. patient or donor signalment, blood results, imaging findings, prior antibiotic treatment) on any outcome measures.

## DISCUSSION

This study failed to find a benefit of adjunctive FMT compared to standard therapy, in terms of faecal score, CIBDAI or rates of owner‐reported clinical improvement, at any timepoint up to 90 days post‐treatment. Unfortunately, due to slow case recruitment (exacerbated by the COVID pandemic at the outset of the study), target sample size was not reached, raising the possibility of a type II error. These findings mirror results of a previous randomised control trial comparing FMT *via* enema with placebo (Collier et al., 2022). Further comparative studies with larger case numbers, or a meta‐analysis of published studies, are warranted to determine if there is an influence of FMT by this route in dogs with chronic enteropathy. No dogs experienced significant adverse effects attributable to treatment during this study. Previous studies in dogs and in humans have reported rare and mostly mild adverse events (Imdad et al., 2023; Winston et al., 2024). However, some adverse effects (pyrexia or abdominal pain) may not have been detected or reported to the case clinician. No new safety concerns associated with FMT were identified in this study, although a recent case series in cats reported a variety of mild to moderate adverse events (Lee et al., 2025).

The broad inclusion criteria employed in this study led to inclusion of a heterogeneous population. Gastrointestinal histopathology was not evaluated, so the presence and degree of inflammatory change, or the presence of neoplastic infiltration were unknown. Other studies have positioned FMT at a later stage in the therapeutic algorithm (*e.g*. for management of dogs already demonstrated to be refractive to diet manipulation (Vecchiato et al., 2025) or with NRE enteropathy (Toresson et al., 2023)). It is possible that a more granular/targeted selection of FMT candidates could be associated with an improved response rate (Toresson et al., 2025).

The rates of owner‐defined clinical improvement recorded by Day 90 in both the FMT (76%, CI 54% to 90%) and standard treatment (73%, CI 40% to 92%) groups here are similar to the 72% success rate reported following a budget‐limited therapeutic approach (diet manipulation, anthelmintics and antibiotics) to chronic enteropathy (Bryan et al., 2019) and the 73% of dogs retrospectively classified as FRE in a study incorporating long‐term follow‐up (Hodel et al., 2024). Since a majority of dogs with chronic enteropathy are anticipated to respond to diet manipulation (Dandrieux, 2016; Dupouy‐Manescau et al., 2024), it is possible that adjunctive benefits conferred by FMT are limited and may be overlooked in underpowered studies. Attribution of causation is difficult in studies without a comparator control group. The favourable outcomes observed following FMT (typically multiple administrations) in observational cohort studies (Bottero et al., 2017; Hodel et al., 2024; Innocente et al., 2022; Niina et al., 2021; Toresson et al., 2023) must be interpreted with caution, as the clinical improvement may be multifactorial.

Optimal protocols for the administration of FMT in dogs have not been established, with no published studies comparing different routes or number of administrations. The use of oral faecal capsules (Brugnoli et al., 2025; Carapeto et al., 2023; Hanifeh et al., 2024; Rojas et al., 2024) to treat dogs with chronic enteropathy can facilitate extended courses of administration. Repeated treatments may also be necessary to elicit a positive response in dogs with refractive chronic enteropathy when administration is *via* rectal enema (Toresson et al., 2025). Higher efficacy was found *via* intrarectal administration in people with recurrent *C. difficile* infection compared to other routes (Gough et al., 2011), and rectal administration was more effective than oral administration for microbiota establishment studies in mouse models (Hanzely et al., 2025).

A systematic review in people with Crohn's disease found no difference in outcome when using fresh or frozen faecal material (Fehily et al., 2021). The use of fresh faeces may be preferred in dogs as repeated freeze–thaw cycles may affect bacterial viability (Saliba et al., 2020), although bacterial diversity was not decreased in samples frozen at −80°C for a year (Barko et al., 2024). While the original protocol for this study was designed in 2019, the methodological approach (FMT sample preparation, processing and administration) employed in this study was largely consistent with the recommendations in the latest international guidelines (Winston et al., 2024). However, the mass of faecal material administered in this study (2 g of faeces/kg bodyweight of recipient) is less than many current studies (typically 5 g of faeces/kg) and may have contributed to the limited clinical change observed. The approach to donor screening was less stringent than described elsewhere (Winston et al., 2024), although the role of *Campylobacter* spp. as an enteropathogen in adult dogs remains uncertain (Marks et al., 2011).

The use of sedation with the sole intent of administering FMT may be considered controversial; two thirds of Companion Animal FMT Consortium members report never or rarely using sedation in dogs receiving FMT (Winston et al., 2024). In this study, FMT administration was timed to occur at the end of a pre‐planned imaging procedure for which the animal had been sedated anyway. This approach ensured consistency across the different treatment groups and meant that all animals were eligible for FMT regardless of their temperament.

Evaluation of response to any intervention requires reliable and repeatable outcome measures. CIBDAI is a clinical scoring system used to evaluate a dog's overall clinical condition based on six elements: attitude/activity, appetite, vomiting, faeces consistency, faeces frequency and weight loss. Each element is scored 0 to 3, where 0 is normal and 3 is a severe change from normal. The CIBDAI scoring system was selected for use in this study as it has been used previously to objectify owner‐reported outcomes (Dye et al., 2013). However, the phrasing of the questionnaire may have caused confusion among some respondents in this study. Several dog owners described persistent diarrhoea as representing no change (CIBDAI score 0) since they were relating the clinical signs to their dog's baseline at the start of the study rather than comparing to a hypothesised normal dog. A thorough explanation of the scoring system at discharge was introduced to address this issue. The mild nature of most cases in this study (low median CIBDAI) at presentation was likely a reflection of the case inclusion criteria. For example, dogs were excluded if their serum albumin suggested possible protein‐losing enteropathy or if there was any prior use of corticosteroids. Hypoalbuminaemia has been identified as a negative prognostic indicator in dogs with chronic enteropathy (Craven et al., 2004). Hypocobalaminaemia is common in dogs with chronic enteropathy and is attributed to distal small intestinal malabsorption and/or small intestinal dysbiosis (Heilmann & Steiner, 2018). Subnormal or low normal serum cobalamin concentrations (<400 ng/L) that would warrant supplementation (Toresson et al., 2016) were found in only 7/42 dogs in this study.

Recruiting dogs based primarily on the presence of chronic diarrhoea may have reduced the sensitivity of the CIBDAI to detect changes over time and, in particular, to detect small differences between groups. Attribute scores for vomiting, weight loss and attitude (Fig 1) contributed relatively little to the variance in CIBDAI; faecal score may represent a more appropriate primary outcome in this patient population. Determining outcomes that matter most to pet owners could also guide objective evaluation of these interventions (Jessen et al., 2024). Arguably, a percentage change in CIBDAI may have represented a more appropriate criterion for evaluating outcome and categorising responders, especially given the typically milder clinical presentation of enrolled dogs. However, the 2‐point change in CIBDAI was defined a priori as the intended outcome measure.

The small sample size also prohibited further comparison of the different donors, with no opportunity to tailor donor selection by breed, age, bodyweight or diet. Whether such factors would influence donor compatibility and clinical efficacy remains uncertain and further study of the microbial composition of donors is required to fully understand the mechanistic action of FMT. A single donor from a study in people with ulcerative colitis was responsible for seven of the nine recipients that achieved clinical remission (Moayyedi et al., 2015). More research is needed to define donor characteristics associated with a greater clinical response to FMT in both people (Lopetuso et al., 2023) and dogs. Measurement of the dysbiosis index of FMT donors is recommended (Winston et al., 2024), but was not performed here.

Inclusion criteria were amended to allow inclusion of dogs previously treated with antimicrobials after 1 month to increase the pool of eligible animals. This approach introduced an additional confounding variable that could influence responsiveness to microbiome manipulation. In a previous study, clinical improvement was not affected by prior antibiotic therapy, so this variable may have been less impactful than originally feared (Vecchiato et al., 2025). Enrolled dogs may also have presented with variable baseline dysbiosis (AlShawaqfeh et al., 2017; Félix et al., 2022) that could influence responsiveness to FMT (Toresson et al., 2023, 2025). The use of fresh faecal material imposed a requirement to plan ahead and ensure the availability of donors prior to initial consultation. Such logistic difficulties, alongside a need to adhere strictly to the study protocol, meant that recruitment relied on the commitment of a few clinicians. While efforts were made to standardise processes of faecal collection, transplant material preparation and administration, the involvement of multiple centres may introduce variability. In an effort to design the most robust study possible, dogs were randomly assigned to their treatment group, and owners were blinded to this. However, a consequence of this was that some owners declined participation in the study as they refused to be randomised or blinded to treatment. Unfortunately, records were not kept at all centres as to the reasons for exclusion.

Further limitations in this study include the lack of a consistent diet change and the asymmetric distribution of additional therapies. These differences may have unpredictably impacted clinical improvement and muted the response to FMT. This study did not demonstrate a clear clinical benefit for adjunctive FMT in dogs with mild chronic enteropathy. The similar outcome for both groups supports high rates of food responsiveness among chronic enteropathy dogs. Further investigations are required to determine if adjunctive FMT may still be valuable for a subset of dogs with chronic diarrhoea or in those that do not readily respond to diet change. No adverse effects were observed in dogs treated in this study, suggesting that FMT following the protocol described in the present study can be employed safely. It may represent a preferable option to antibiotic or corticosteroid trials given the established adverse effect profiles of these treatments.

### Author contributions

**M. J. Whittle:** Conceptualization; investigation; writing – original draft; writing – review and editing; validation; methodology; software; formal analysis; project administration; data curation; supervision; resources. **F. Duplan:** Conceptualization; funding acquisition; writing – original draft; validation; formal analysis; writing – review and editing. **M. Trehy:** Conceptualization; writing – original draft; writing – review and editing; methodology; formal analysis. **F. Allerton:** Conceptualization; investigation; writing – original draft; methodology; validation; writing – review and editing; software; formal analysis; project administration; data curation; supervision; resources. **P. Watson:** Conceptualization; writing – original draft; writing – review and editing; methodology; software; supervision. **G. C. A. Amos:** Conceptualization; writing – original draft; writing – review and editing; software; supervision. **S. Borgonovi:** Writing – original draft; writing – review and editing; conceptualization; methodology; formal analysis. **I. Gajanayake:** Conceptualization; writing – original draft; writing – review and editing; methodology; formal analysis; supervision; data curation. **M. Dunning:** Conceptualization; validation; writing – review and editing; writing – original draft; formal analysis; supervision. **H. Swales:** Conceptualization; investigation; writing – original draft; writing – review and editing; methodology; formal analysis. **A. Kent:** Conceptualization; investigation; writing – original draft; writing – review and editing; validation; methodology; formal analysis; data curation; supervision. **G. Pinchbeck:** Conceptualization; writing – original draft; validation; writing – review and editing; formal analysis; supervision. **J. Bazelle:** Conceptualization; investigation; writing – original draft; methodology; writing – review and editing; formal analysis; software; project administration; data curation. **C. Prior:** Conceptualization; investigation; writing – original draft; writing – review and editing; methodology; formal analysis. **T. Sparks:** Conceptualization; investigation; writing – original draft; writing – review and editing; methodology; formal analysis. **K. McCallum:** Conceptualization; investigation; writing – original draft; writing – review and editing; methodology; formal analysis.

### Conflict of interest

Micky Whittle, Luke Durkin, Phil Watson and Greg Amos are employees of Mars Petcare, a manufacturer of pet food and provider of veterinary services. The remaining authors declare no conflict of interest.

## Supporting information


**Fig S1.** Faeces consistency scores according to the WALTHAM™ faeces scoring system.

**Table S1.** Inclusion criteria for dogs entering the study

**Table S2.** Exclusion criteria for dogs entering the study

**Table S3.** List of breeds (and number in each breed) in the standard treatment and FMT groups

**Table S4.** Baseline haematological and biochemical values for the studied dogs

## Data Availability

The data that support the findings of this study are available from the corresponding author upon reasonable request.

## Endnotes

^1^
Veterinary Pathology Group, Farm Business Park, Unit2a, Higham Gobion, Shillington, SG5 3HR.

^2^
WSAVA Body condition score chart: https://wsava.org/wp‐content/uploads/2020/01/Body‐Condition‐Score‐Dog.pdf.

^3^
Waltham faecal scoring chart: accessible from https://www.waltham.com/s3media/2020‐05/waltham‐scoring.pdf and included as Figure S1.
