# Supplementary material for: Clinical benefit of faecal microbiota transplantation administered via a single retention enema as an adjunctive treatment in dogs with chronic enteropathy: a randomised controlled trial
Source: J Small Anim Pract. 2026 Apr 17;67(9):798–809. doi: 10.1111/jsap.70137 (PMC13539667; doi:10.1111/jsap.70137)

Supplementary Materials Figure 1. Faeces consistency scores according to the WALTHAM™ Faeces Scoring System

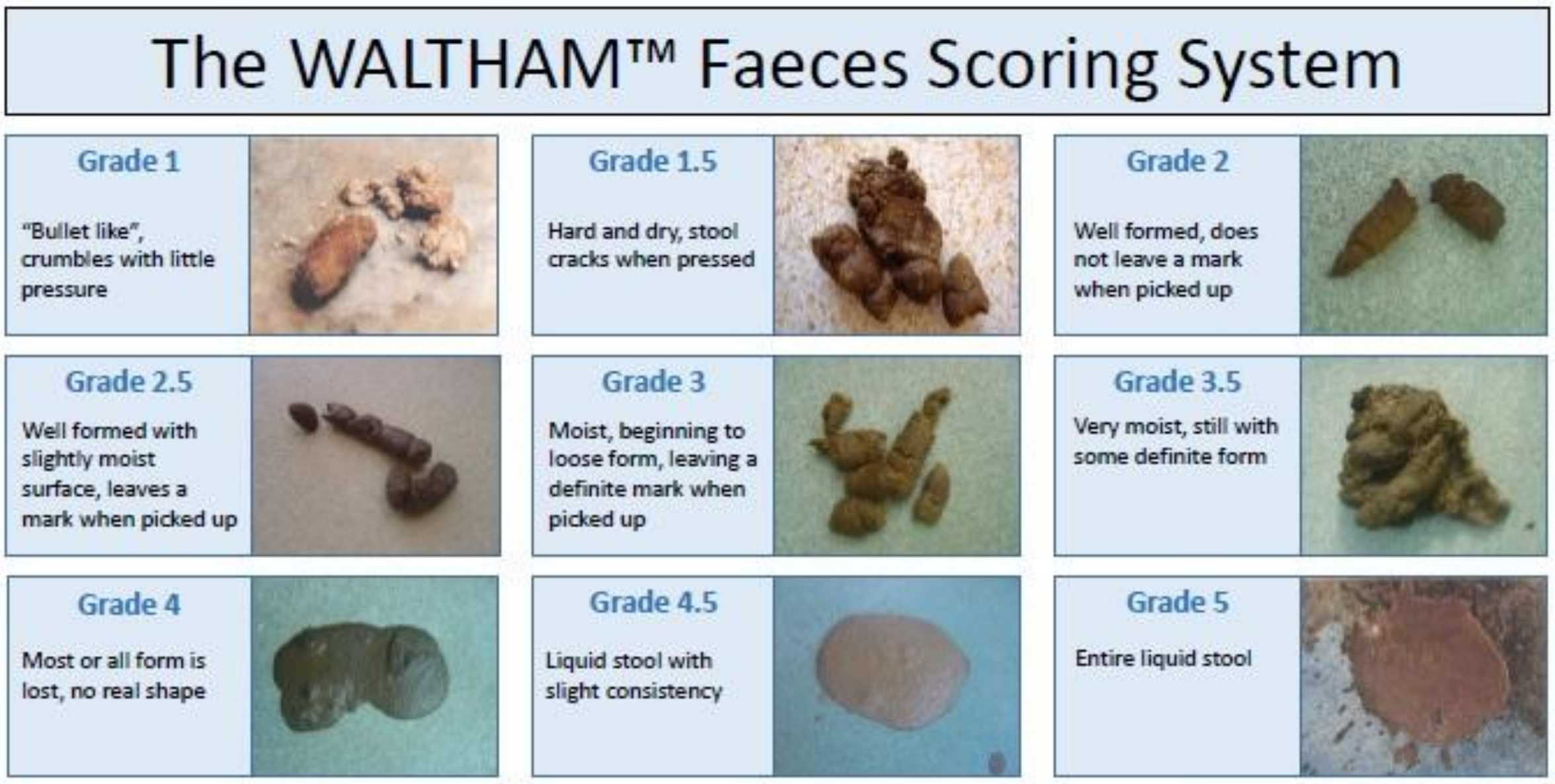

Supplement: Supplementary file 1 [file JSAP-67-798-s001.pdf]
