# Supplementary material for: Clinical benefit of faecal microbiota transplantation administered via a single retention enema as an adjunctive treatment in dogs with chronic enteropathy: a randomised controlled trial
Source: J Small Anim Pract. 2026 Apr 17;67(9):798–809. doi: 10.1111/jsap.70137 (PMC13539667; doi:10.1111/jsap.70137)
Supplement: Supplementary file 2 [file JSAP-67-798-s002.docx]

Supplementary Materials Table 1. Inclusion criteria for dogs entering the study.

| The owner is willing and able to give informed consent for participation of their dog in the trial | ✔ |
| --- | --- |
| The dog is aged 6 months to 10 years | ✔ |
| The dog has a history of chronic (>3 weeks) small intestinal or mixed small/large intestinal diarrhoea | ✔ |
| The dog has a serum albumin ≥ 20g/L | ✔ |
| Hypoadrenocorticism is excluded (either via basal cortisol >55noml/L or a compatible ACTH stimulation test) | ✔ |
| An abdominal ultrasound and/or abdominal CT is performed by a specialist in diagnostic imaging or overseen by a specialist in internal medicine | ✔ |
| The dog is sedated for the imaging procedure | ✔ |
