# Supplementary material for: Clinical benefit of faecal microbiota transplantation administered via a single retention enema as an adjunctive treatment in dogs with chronic enteropathy: a randomised controlled trial
Source: J Small Anim Pract. 2026 Apr 17;67(9):798–809. doi: 10.1111/jsap.70137 (PMC13539667; doi:10.1111/jsap.70137)
Supplement: Supplementary file 3 [file JSAP-67-798-s003.docx]

Supplementary Materials Table 4. Baseline haematological and biochemical values for the studied dogs

| **Parameter** | **Median** | **Units** | **Range** | **Reference interval** | **Number** |
| --- | --- | --- | --- | --- | --- |
| Albumin | 30 | g/L | 20-39 | 24-40 | 42 |
| Total protein | 61 | g/L | 46-76 | 52-77 | 41 |
| Cholesterol | 5.7 | mmol/L | 2.2-11.9 | 3.8-7.0 | 40 |
| Creatinine | 83 | μmol/L | 51-156 | 40-125 | 41 |
| Total bilirubin | 3 | μmol/L | 1.8-9 | 0-9 | 41 |
| Alanine transferase | 46 | U/L | 23-154 | 0-40 | 41 |
| Haematocrit | 48 | % | 31-65 | 37-55 | 41 |
| Leucocyte count | 10.2 | ×10^9^/L | 3.8-18.7 | 6.0-15.0 | 41 |
| Neutrophil count | 7.2 | ×10^9^/L | 2.6-13.3 | 3.0-11.5 | 41 |
| Lymphocyte count | 2.0 | ×10^9^/L | 0.7-5.2 | 1.0-4.8 | 41 |
| Eosinophil count | 0.5 | ×10^9^/L | 0.0-3.6 | 0.0-1.2 | 41 |
| Cobalamin | 522 | ng/L | 164-1655 | 251-908 | 40 |
| Folate | 13.4 | μg/L | 5.5-53.5 | 7.7-24.4 | 30 |
| TLI | 28.0 | μg/L | 3.5-51 | 5-40 | 26 |
| DGGR lipase | 24.0 | U/L | 7-532 | 0-50 | 32 |
| cPLI | 57.0 | μg/L | NA | <200 | 1 |
