# Supplementary material for: Clinical benefit of faecal microbiota transplantation administered via a single retention enema as an adjunctive treatment in dogs with chronic enteropathy: a randomised controlled trial
Source: J Small Anim Pract. 2026 Apr 17;67(9):798–809. doi: 10.1111/jsap.70137 (PMC13539667; doi:10.1111/jsap.70137)
Supplement: Supplementary file 4 [file JSAP-67-798-s004.docx]

Supplementary Materials Table 3. List of breeds (and number in each breed) in the standard treatment and FMT groups:

| **Standard treatment group** | **Number** |
| --- | --- |
| Cross breed | 5 |
| Labrador Retriever | 2 |
| Cocker spaniel | 1 |
| English Springer Spaniel | 1 |
| Shih Tzu | 1 |
| German Shepherd | 1 |
| Weimaraner | 1 |
| Cavalier King Charles Spaniel | 1 |
| Miniature Schnauzer | 1 |
| Boxer | 1 |
| Bichon Frise | 1 |
| Staffordshire Bull Terrier | 1 |
|  |  |
| **FMT group** | **Number** |
| Cross breed | 8 |
| German Shepherd | 3 |
| Hungarian Vizsla | 2 |
| Golden Retriever | 2 |
| Cocker spaniel | 1 |
| English Springer Spaniel | 1 |
| Greyhound | 1 |
| Collie | 1 |
| Dachshund | 1 |
| Border Collie | 1 |
| Maltese | 1 |
| Miniature Schnauzer | 1 |
| Boxer | 1 |
| Kerry Blue Terrier | 1 |
