# Supplementary material for: Clinical benefit of faecal microbiota transplantation administered via a single retention enema as an adjunctive treatment in dogs with chronic enteropathy: a randomised controlled trial
Source: J Small Anim Pract. 2026 Apr 17;67(9):798–809. doi: 10.1111/jsap.70137 (PMC13539667; doi:10.1111/jsap.70137)
Supplement: Supplementary file 5 [file JSAP-67-798-s005.docx]

Supplementary Materials Table 2. Exclusion criteria for dogs entering the study.

| Owners are unable to provide informed consent or unwilling to be blinded to treatment group | ✘ |
| --- | --- |
| The dog is aged <6 months or > 10 years | ✘ |
| The dog cannot be or is not sedated for abdominal imaging | ✘ |
| There is evidence of an extra-intestinal cause of diarrhoea or exclusively large-intestinal diarrhoea | ✘ |
| The dog has hypoalbuminemia (<20 g/L) | ✘ |
| The dog is receiving probiotics at the start of the trial | ✘ |
| The dog has received anti-inflammatories (prednisolone) or other immunosuppressants within four weeks of enrolment onto the trial | ✘ |
| The dog has received antibiotics within four weeks of enrolment onto the trial | ✘^*^ |

Note the final exclusion criterion* was removed after one month of the trial.
